# Correlation between air pollution and prevalence of conjunctivitis in South Korea using analysis of public big data

**DOI:** 10.1038/s41598-022-13344-5

**Published:** 2022-06-16

**Authors:** Sanghyu Nam, Mi Young Shin, Jung Yeob Han, Su Young Moon, Jae Yong Kim, Hungwon Tchah, Hun Lee

**Affiliations:** 1 Department of Ophthalmology, Asan Medical Center, University of Ulsan College of Medicine, Seoul, Korea; 2 Graduate School of Public Health, Catholic University of Korea, Seoul, Korea; 3 Seoul Metropolitan Office of Education, Seoul, Korea

**Keywords:** Environmental sciences, Health care, Risk factors

## Abstract

This study investigated how changes in weather factors affect the prevalence of conjunctivitis using public big data in South Korea. A total of 1,428 public big data entries from January 2013 to December 2019 were collected. Disease data and basic climate/air pollutant concentration records were collected from nationally provided big data. Meteorological factors affecting eye diseases were identified using multiple linear regression and machine learning analysis methods such as extreme gradient boosting (XGBoost), decision tree, and random forest. The prediction model with the best performance was XGBoost (1.180), followed by multiple regression (1.195), random forest (1.206), and decision tree (1.544) when using root mean square error (RMSE) values. With the XGBoost model, province was the most important variable (0.352), followed by month (0.289) and carbon monoxide exposure (0.133). Other air pollutants including sulfur dioxide, PM_10_, nitrogen dioxides, and ozone showed low associations with conjunctivitis. We identified factors associated with conjunctivitis using traditional multiple regression analysis and machine learning techniques. Regional factors were important for the prevalence of conjunctivitis as well as the atmosphere and air quality factors.

## Introduction

Conjunctivitis is as commonly presenting disease at ophthalmology clinics, caused mainly by viral infections, allergic reactions, or atopy. Environmental factors have also been implicated in incidences of conjunctivitis^1^. Consistent contact with the ocular surface in eyes allows toxins to directly access ocular structures and cause conjunctivitis-like symptoms^2^. Additionally, the effects of environmental pollution on human health can vary depending on the composition of and degree and time of exposure to air pollutants^3,4^.

Previous studies have focused on evaluating the association between air pollution and health problems related to respiratory organs and cardiovascular vessels^5,6^. However, air quality can affect not only the respiratory and cardiovascular systems, but also the ocular surface of the eye, with which air comes into direct contact. Air pollutants such as ozone, nitrogen dioxide, and sulfur dioxide have been associated with conjunctivitis^7^. Furthermore, one study found relationships between the levels of particulate matter with aerodynamic diameter < 10 μm (PM_10_) and emergency room visits for keratoconjunctivitis, ischemic heart disease, and stroke in Korea^8^. For medical and health care for conjunctivitis, studies on the prevalence of ocular surface disease like keratoconjunctivitis need to be conducted to report the relationship between the prevalence of conjunctivitis and air and atmosphere quality, as well as population factors such as region, number of people, age, and gender. However, there has been no nationwide study evaluating the relationship between various air pollutants and conjunctivitis.

Therefore, in the present study, we investigated how changes in weather and population factors can affect the prevalence of conjunctivitis using public big data provided by various Korean governmental institutions. Furthermore, we determined whether air pollution increases the risk of conjunctivitis by using machine learning prevalence prediction models.

## Results

**Figure 1:**
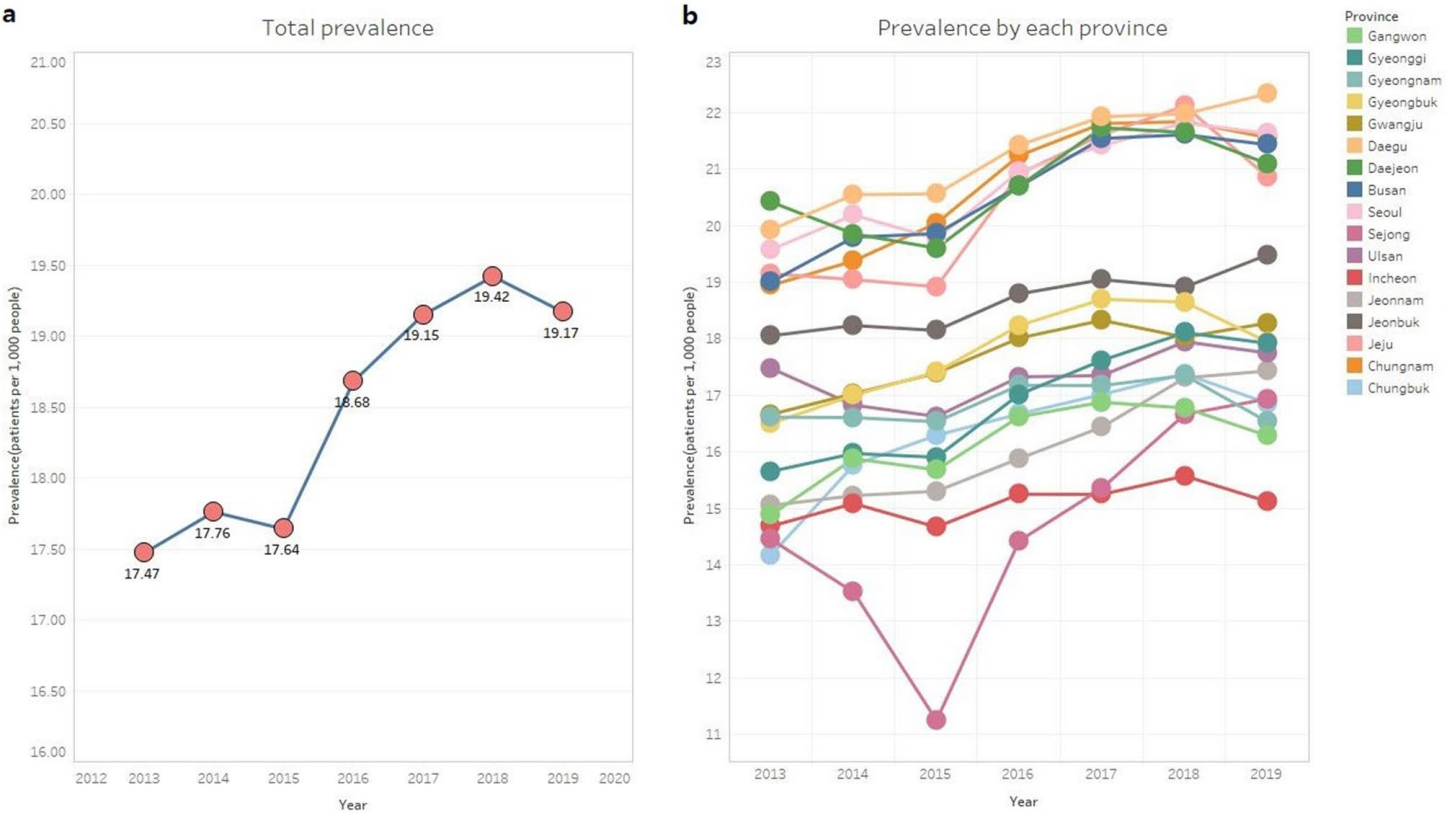
Prevalence of conjunctivitis. (**a**) Prevalence of conjunctivitis by year (number of patients per 1,000 people). The number of patients increased from 2013 to 2019. (**b**) Prevalence by each province.

According to the annual prevalence trends, 19.17 patients per 1,000 people were diagnosed with conjunctivitis in 2019 compared to 17.47 patients per 1,000 people in 2013. The number of patients per year tended to increase from 2013 to 2019, with a mild decrease in 2015 and 2019 (Fig. 1a). Prevalence by each province also showed a steady upward curve. In some regions, the prevalence decreased in 2015, but increased again from 2016 (Fig. 1b).

**Figure 2:**
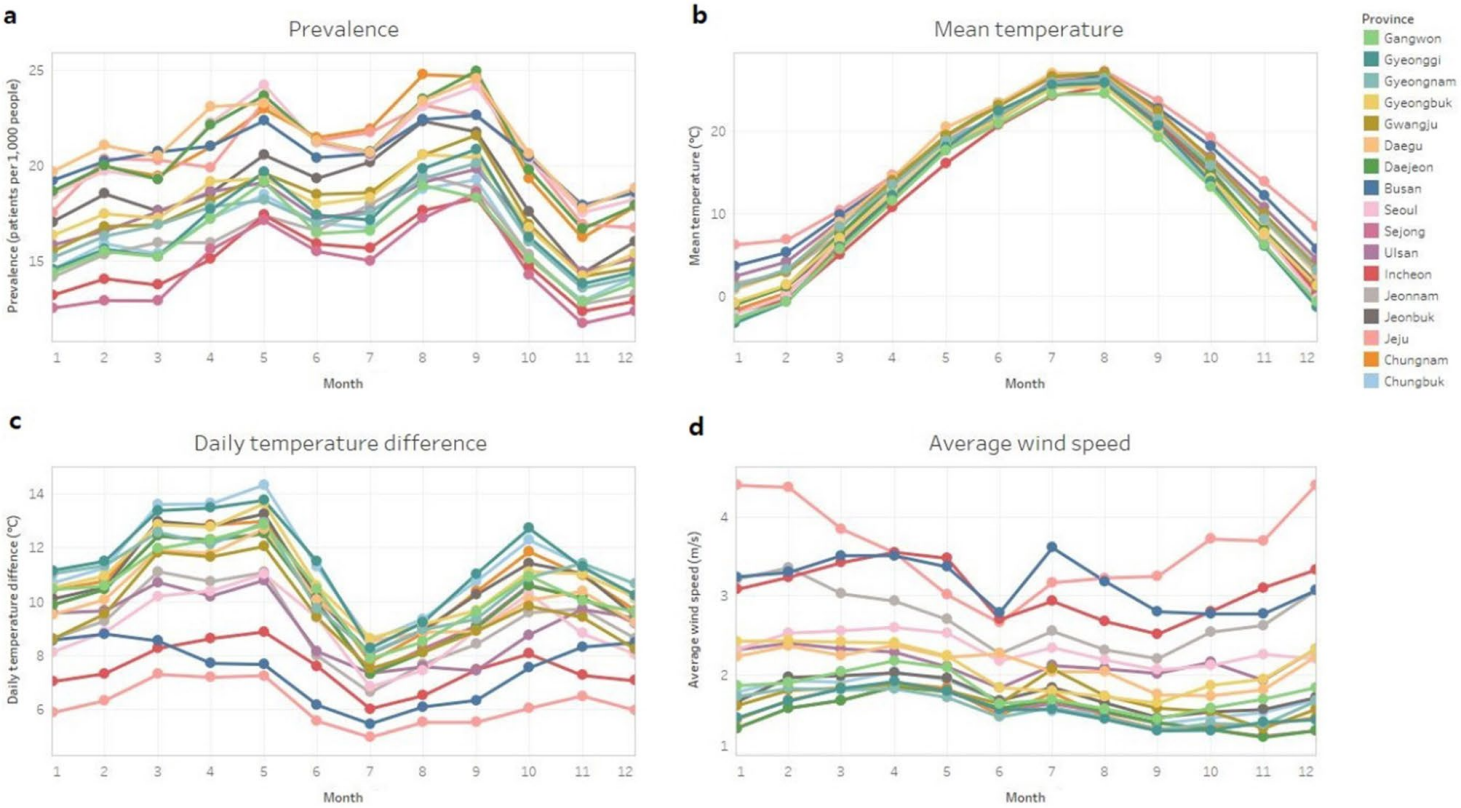
Prevalence of conjunctivitis and weather parameters by month in each region. (**a**) Prevalence of conjunctivitis, (**b**) mean temperature, (**c**) mean daily temperature difference, and (**d**) mean wind speed.

Figure 2 shows the prevalence of conjunctivitis and weather parameters by month in each region of Korea. The monthly prevalence of conjunctivitis peaked in May and September in all provinces (Fig. 2a). The prevalence tended to increase as winter changed to summer with peaks between seasons. The mean temperature was highest in July and August and lowest in January and February (Fig. 2b). All regions showed similar trends in the mean temperature. The daily temperature difference was highest in spring and fall, but some provinces, including Jeju Island, Busan City, and Incheon City, showed smaller temperature differences because they were coastal areas (Fig. 2c). The average wind speed did not show much change by month; only in the winter season in Jeju Island, relatively high wind speeds were observed compared to those in the other provinces (Fig. 2d).

**Figure 3:**
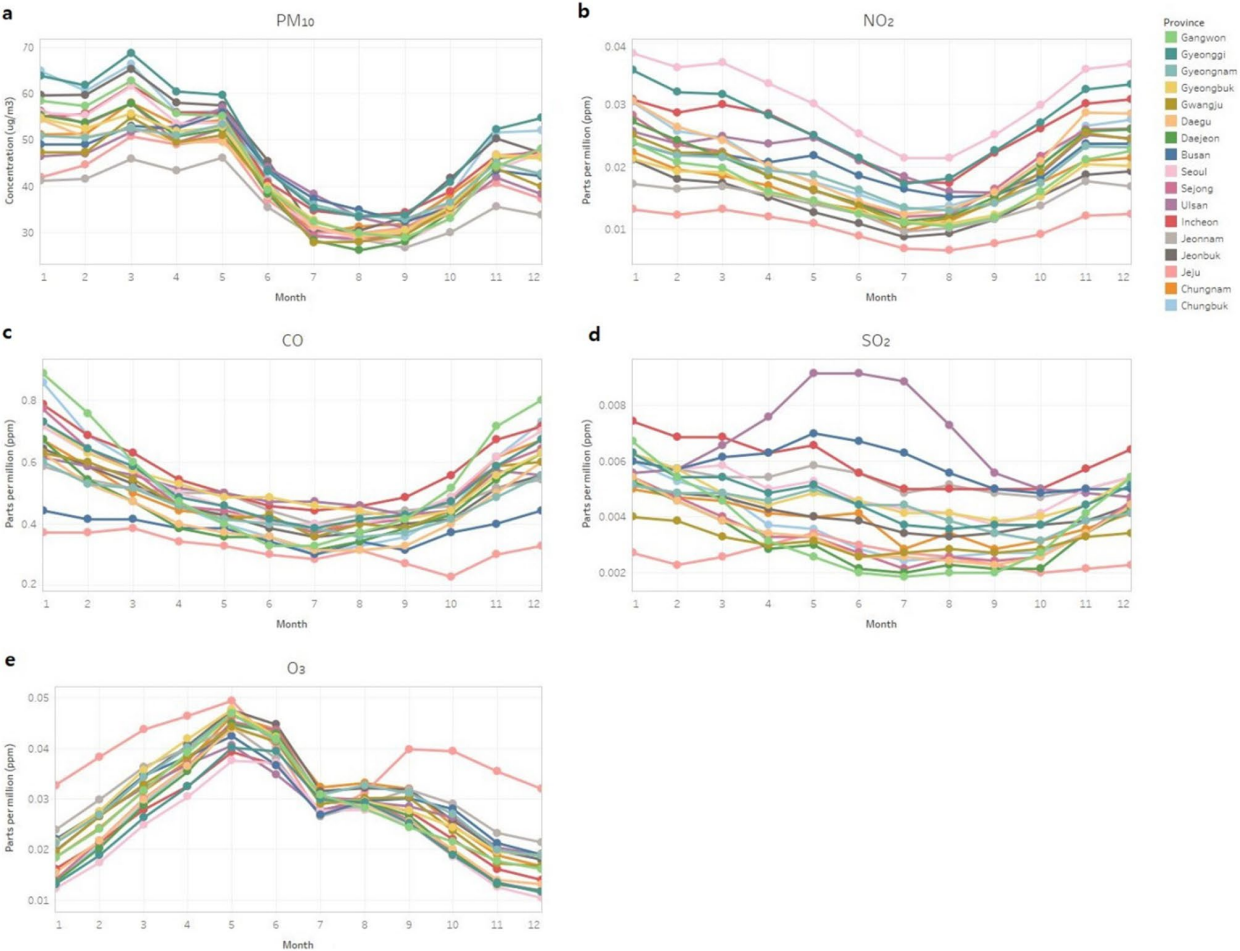
Air quality parameters by month in each region. (**a**) concentration of sulfur dioxide, (**b**) concentration of nitrogen dioxide, (**c**) concentration of carbon monoxide, (**d**) concentration of PM_10_, and (**e**) concentration of ozone.

Figure 3 shows monthly air quality data by region. In all provinces, PM_10_ levels remained high from winter to spring, decreased starting in May with the lowest levels in August, and increased again to high levels from September to spring (Fig. 3a). Other air quality variables including concentrations of nitrogen dioxide, carbon monoxide, and sulfur dioxide, showed low levels in summer and high levels in winter (Fig. 3b–d). Levels of sulfur dioxide were especially unique in Ulsan city and highest in summer (Fig. 3d). This result may be because Ulsan city is heavily industrialized. The concentration of ozone was highest in spring and decreased from summer through winter in all provinces (Fig. 3e).

Pearson’s correlation coefficient analysis was performed to evaluate the relationship between the prevalence of conjunctivitis and independent variables (Table 1). The results showed positive correlations with average temperature, humidity, precipitation, and ozone concentrations; negative correlations were described for daily temperature differences, average wind speeds, and concentrations of sulfur dioxide, nitrogen dioxide, carbon monoxide, and PM_10_.

**Table 1:** Correlation coefficients of variables using correlation analysis between prevalence and temperature or air quality parameters. Average temperature, humidity, precipitation, and ozone showed positive correlation, daily temperature difference, average wind speed, sulfur dioxide, nitrogen dioxide, carbon monoxide and PM_10_ showed negative correlation.

	1	2	3	4	5	6	7	8	9	10	11
1. Regional Prevalence		**0.52**	-**0.11**	**0.13**	**0.29**	0.04	-**0.28**	-**0.37**	**0.43**	-**0.56**	-**0.26**
2. Temperature	**0.52**		-**0.26**	**0.61**	**0.61**	-**0.07**	-**0.33**	-**0.64**	**0.54**	-**0.74**	-**0.58**
3. Daily temperature difference	**-0.11**	-**0.26**		-**0.50**	**-0.43**	-**0.45**	-0.01	**0.22**	**0.16**	**0.23**	**0.48**
4. Humidity	**0.13**	**0.61**	-**0.50**		**0.59**	-**0.16**	-**0.27**	-**0.55**	**0.05**	-**0.39**	-**0.58**
5. Precipitation	**0.29**	**0.61**	-**0.43**	**0.59**		0.01	-**0.25**	-**0.49**	**0.21**	-**0.48**	-**0.49**
6. Wind speed	0.04	-**0.07**	-**0.45**	**-0.16**	0.01		**0.25**	-0.04	**0.21**	-**0.13**	**0.07**
7. SO_2_	-**0.28**	-**0.33**	-0.01	-**0.27**	-**0.25**	**0.25**		**0.51**	-**0.18**	**0.50**	**0.43**
8. NO_2_	**-0.37**	-**0.64**	**0.22**	-**0.55**	-**0.49**	-0.04	**0.51**		-**0.55**	**0.67**	**0.55**
9. O_3_	**0.43**	**0.54**	**0.16**	**0.05**	**0.21**	**0.21**	-**0.18**	-**0.55**		-**0.55**	0.00
10. CO	-**0.56**	-**0.74**	**0.23**	-**0.39**	-**0.48**	-**0.13**	**0.50**	**0.67**	-**0.55**		**0.53**
11. PM_10_	-**0.26**	-**0.58**	**0.48**	-**0.58**	-**0.49**	**0.07**	**0.43**	**0.55**	0.00	**0.53**	

SO_2_, sulfur dioxide; NO_2_, nitrogen dioxide; O_3_, Ozone; CO, carbon monoxide; PM_10_, levels of particulate matter with aerodynamic diameter < 10 μm.

Significant values are in bold.

In the multiple regression analysis, the coefficient of determination was 0.8789. Based on the high predictive power of the multiple regression analysis, we assessed the best performance prevalence prediction model among machine learning techniques including extreme gradient boosting (XGBoost), decision trees, and random forest. The outcome performances of each model were compared using root mean square error (RMSE), and the training and test set ratio was 9:1. As a result, model performance was shown in the order of XGBoost (1.180), multiple regression (1.195), random forest (1.206), and decision tree (1.544) (Table 2).

**Table 2:** Comparison of modeling techniques on root mean square error values.

Model	RMSE
Multiple linear regression	1.195
XGBoost	1.180
Random forest	1.206
Decision tree	1.544

RMSE, root mean square error.

**Figure 4:**
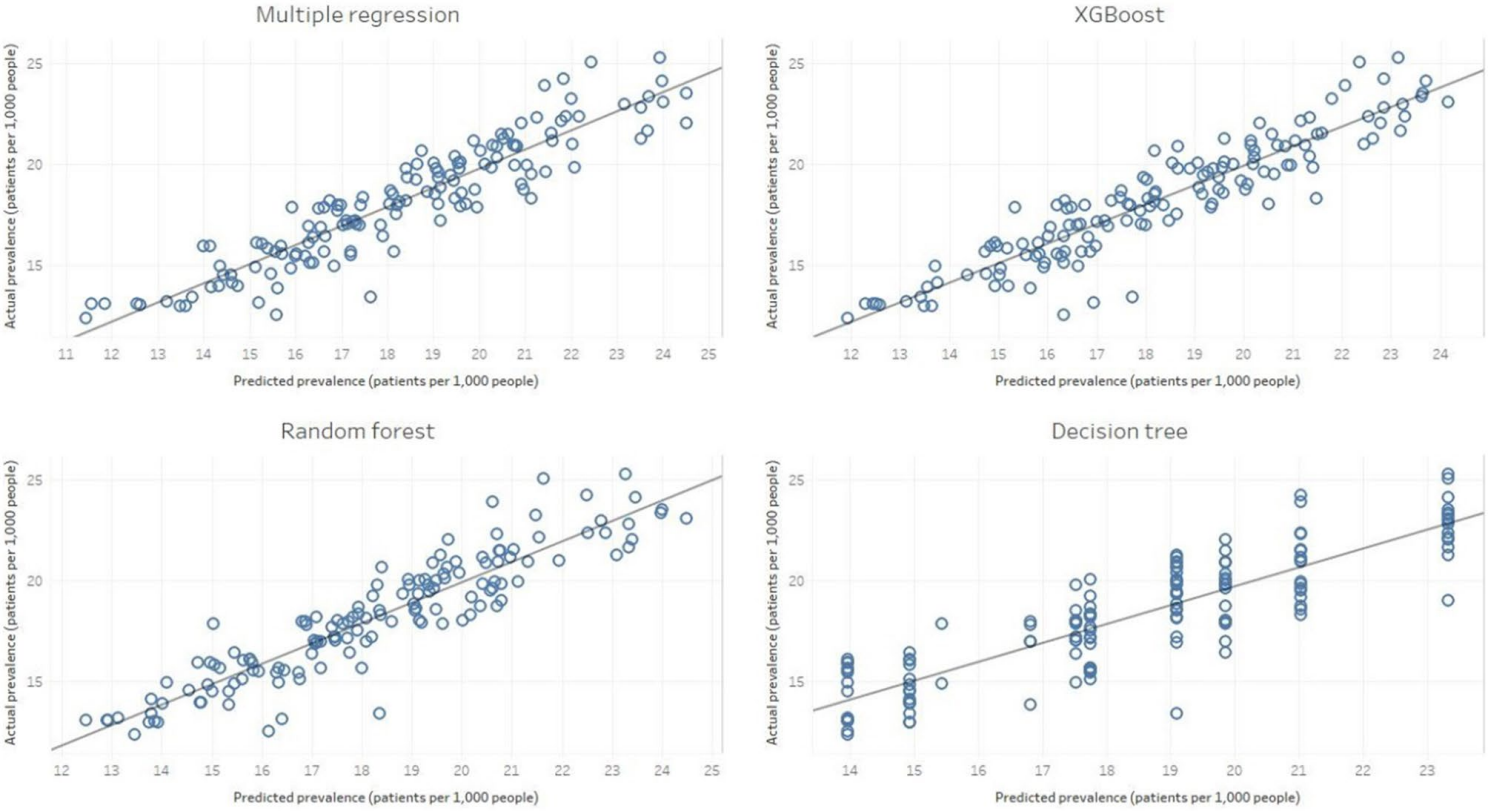
Predicted prevalence to actual prevalence for each model. The XGBoost model shows the most accurate prediction model and the decision tree model shows the least accurate prediction.

According to the scatterplots showing the difference between real and predictive values in machine learning predictions, XGBoost's predictions were best suited to real values. The decision trees had the lowest fit among the other models, similar to that in previous studies (Fig. 4)^9^. Based on the results from XGBoost prediction model, which had the best predictive power, the most important variables were province (gain value: 0.352), month (0.289), and carbon monoxide level (0.133; Table 3).

**Table 3:** Variables of importance in the XGBoost prediction model.

Variable	Gain
Province (local)	0.352
Month	0.289
CO	0.133
Temperature	0.060
Humidity	0.030
Wind speed	0.030
Precipitation	0.022
Temperature fluctuation	0.021
O_3_	0.019
PM_10_	0.017
SO_2_	0.016
NO_2_	0.013

CO, carbon monoxide; O_3_, Ozone; PM_10_, levels of particulate matter with aerodynamic diameter < 10 μm; SO_2_, sulfur dioxide; NO_2_, nitrogen dioxide.

## Discussion

In the present study, based on countrywide public big data, we evaluated the effects of weather and air quality variables on the prevalence of conjunctivitis and compared the performance of predictive modeling. There have been previous studies on correlations between air pollution and various diseases, such as keratoconjunctivitis, ischemic heart disease, stroke, and respiratory diseases^8,10^. Although there are various datasets relating to eye diseases, it is well known that the ocular surface, including the cornea, is always exposed to the air, and subsequently, symptoms of conjunctivitis and air pollutants are always associated^11^. Therefore, in this study, we selected ocular surface diseases, such as keratoconjunctivitis, conjunctivitis, and blepharoconjunctivitis, to analyze their association with environmental factors.

The prevalence of conjunctivitis showed an increasing trend from 2013 to 2019. Based on a monthly analysis, the prevalence was the highest during spring and fall with two peaks in May and September and was the lowest in winter. This finding aligns with those of a previous study^12^, during which the prevalence of allergic conjunctivitis increased from spring to fall in accordance with other increased allergen levels such as those of dust and pollen.

Among the predictive models, the XGBoost model showed the best performance, followed by multiple regression analysis, random forest, and decision tree modeling. The most important variable according to the XGBoost model was province, followed by month and carbon monoxide level. Notably, region was not estimated as an effective factor in a previous study conducted in Korea^8^. This difference may be attributed to different climatic factors, air quality factors, and medical systems in each province. We believe that further research on regional prediction models is necessary.

The second most important factor was the month of the year. As previously mentioned, prevalence differed from month to month with higher rates during the spring and fall. It is notable that the monthly impact was greater than the impact of other climatic or air quality factors. These climatic and air quality factors are comprehensively reflected in each monthly period. Therefore, considering the month as a sole factor, it may be most important compared to other climatic and air quality factors because it can predict the characteristics of the climate and air quality itself.

Among air pollutants, carbon monoxide was most highly associated with the prevalence of conjunctivitis (0.133) when compared to the associations of sulfur dioxide (0.016), PM_10_ (0.017), nitrogen dioxide (0.013), and ozone (0.019). A few previous studies have shown that carbon monoxide has minor effects on the prevalence of conjunctivitis. One report showed an association between carbon monoxide levels and emergency room visits for asthma^10^, and another reported a positive association between carbon monoxide levels and the prevalence of conjunctivitis^13^. In contrast, Chang et al. reported that carbon monoxide had only a non-significant influence on nonspecific conjunctivitis cases in outpatient visits, due to the absence of ocular irritation as a consequence of carbon monoxide exposure^14^. According to our study, conjunctivitis and carbon monoxide were negatively correlated, and to our knowledge, it is the only study that has shown negative correlation results. We believe that increases in carbon monoxide levels are closely related to increased use of fuels for heating during cold seasons. The concentrations of carbon monoxide decrease during the summer and increase in the winter. Our results showed that concentrations of carbon monoxide remain low from April to September and then increase from October to March. The prevalence of conjunctivitis begins to increase in April, peaks in May and September, and decreases from October to March. This change is thought to be the result of similarity in monthly trends rather than a direct association between carbon monoxide and conjunctivitis.

PM_10_ is a complex component comprised of metal compounds such as nickel, aluminum, silicon, and titanium dioxide, which are correlated with ocular symptoms^15^. Lu et al. reported that PM_10_ is associated with conjunctivitis^16^, but another study found no association between the two^14^. Automobile exhaust is the main source of atmospheric sulfur dioxide and nitrogen dioxide^17^. One Brazilian study found a clear dose–response relationship between the nitrogen dioxide level and goblet cell hyperplasia, suggesting morphological changes in the conjunctival epithelium as an adaptive response to chronic environmental injury^18^. Sulfur dioxide was significantly associated with conjunctivitis during outpatient hospital and emergency room visits^13,19^.

Ozone is an important factor in ‘‘summer smog,’’ generated at ground level by photochemical reactions involving ultraviolet radiation within the atmospheric mixture of nitrogen oxide and hydrocarbons derived from vehicular emissions. Atmospheric concentrations of ozone and nitrogen oxide have been linked to asthma and other airway inflammatory diseases^20,21^. Ozone can induce an inflammatory response in the ocular surfaces in mouse models and in cultured human conjunctival epithelial cells^22^. Moreover, exposure to ozone exacerbates the detrimental effects on the integrity of the ocular surface, caused by conjunctival allergic reactions and further increases the inflammatory response^23^.

The results of correlations between conjunctivitis and air pollutants are inconsistent. Fu et al.^13^ revealed a significant risk of nitrogen dioxide for the prevalence of conjunctivitis, while Jamaludin et al.^24^ did not. With regard to PM_10_, Chang et al.^14^ revealed PM_10_ to be significantly associated with conjunctivitis risk. However, in a different study conducted by Chiang et al.^7^, nitrogen dioxide had no significant effect on the risk of conjunctivitis. Fu et al.^13^ revealed that the correlation between sulfur dioxide and conjunctivitis risk was significant. Previous meta-analyses of five air pollutants (PM_10_, sulfur dioxide, carbon monoxide, nitrogen dioxide, and ozone) showed a positive correlation between these pollutants and conjunctivitis^25^. We propose that the contradictory results may be attributable to the study design. Our results are different from those of previous studies, with carbon monoxide being negatively correlated with conjunctivitis. This finding is believed to be due to the slight difference in the analyses methods and origins of data relating to climatic factors and air quality.

In this study, administrative district demographics, weather data, air quality data, and disease data were collected; research was conducted after pre-processing data for effective use and statistical analysis. Machine learning techniques allow users to form guidelines and create new insights using public data. Although ecological analysis has limitations in application to individuals, this study allowed us to obtain individual diagnostic data and variables for subsequent research into weather factors and predictive models for eye disease.

Our study had some limitations. First, the information regarding actual clinical examinations was unavailable in the claims data. Biological factors other than ambient air quality that can cause eye diseases were also undetermined. The International Standard Disease Classification (ICD-10) diagnoses may not be precise enough to reflect the true etiology of conjunctival disease. Additionally, this study used second-hand data to evaluate associations between environmental exposures and diseases; we assumed that the participants were exposed to the same levels of air pollutants as reflected in the measurements of their residential regions. Thus, it is possible that the risk was underestimated^26^.

In conclusion, we demonstrated associations between weather factors and the prevalence of conjunctivitis via large-scale analyses of nationally provided big data. Traditional multiple regression analysis and machine learning techniques were used to identify the best prediction model. With the best prediction performance by the XGBoost model, region (province), month, and carbon monoxide concentration were found to be the important variables contributing to the prevalence of conjunctivitis. It is meaningful that the association of carbon monoxide among air pollutants was high, and it is also important that regional and monthly factors were related to conjunctivitis along with air quality factors. Consideration of these variables would be helpful for detection and management of conjunctivitis in the clinical field.

## Methods

### Study object and data source

This study used information from health insurance claims obtained by the Korean Statistical Information Service (KOSIS) and daily meteorological records from the Korea Meteorological Administration (KMA) and the Korea Environment Corporation (Air Korea). The KOSIS provided data from 17 provinces including data on population by province. Basic climate data from the KMA included monthly 24-h weather data regarding average temperature, highest and lowest temperatures, relative humidity, rainfall, and wind speeds. Air Korea provided climate data including concentrations of PM_10_, nitrogen dioxide, sulfur dioxide, carbon monoxide, and ozone. The ambient PM_10_ concentration was measured by total 600 air quality monitoring networks, urban air monitoring networks (495), national background concentration networks (11), suburban air monitoring networks (27), road-side air monitoring networks (52), and port air monitoring networks (15). All subjects were assumed to be exposed to the same levels of air pollutants as measured by permanent weather monitoring. The National Ambient Air Quality Standards of South Korea provided by the National Institute of Environmental Research are added in Supplementary Table 1.

Categories of eye diseases were defined using the ICD-10 and collected using the Health Insurance Review and Assessment Service (HIRA)^27,28^. Disease categories were allergic conjunctivitis, acute conjunctivitis, chronic conjunctivitis, lacrimal gland disorders, blepharoconjunctivitis, keratoconjunctivitis and other unspecific conjunctivitis. Cases of infectious conjunctivitis from pathogens such as adenovirus, herpes virus, meningococcus, gonococcus, acanthamoeba, and trachoma and other bacterial conjunctivitis were excluded. The number of patients diagnosed with the disease was counted and converted to the regional prevalence using local population counts, which was set as a dependent variable.

Administrative district demographics, meteorological data, air quality data, and disease data were collected from January 2013 to December 2019. All variables analyzed are presented in Table 4, and data pre-processing was conducted for effective data use and statistical analysis. The Institutional Review Board of Asan Medical Center (University of Ulsan College of Medicine) instead of approved the waiver of reviewing this study (2021-0173). This study was conducted according to the ethical principles outlined in the Declaration of Helsinki. The requirement for obtaining informed consent was waived.

**Table 4:** Basic variables from government-provided big data.

Variable	Description	Data Source
Province*	17 provinces (Si, Do)	KOSIS
Population	Population by province	KOSIS
Temperature (℃)	Mean temperature by province, round the number to 2 places	KMA
Highest temperature (℃)	Mean highest temperature by province, round the number to 2 places	KMA
Lowest temperature (℃)	Mean lowest temperature by province, round the number to 2 places	KMA
Temperature difference (℃)	Mean daily temperature difference by province, round the number to 2 places	KMA
Humidity (%)	Mean relative humidity by province, round the number to 2 places	KMA
Precipitation (mm)	Monthly total precipitation by province, round the number to 2 places	KMA
Wind speed (m/s)	Mean wind speed by province, round the number to 2 places	KMA
SO_2_ (ppm)	Concentration of SO_2_ by province, round the number to 4 places	Air Korea
NO_2_ (ppm)	Concentration of NO_2_ by province, round the number to 4 places	Air Korea
O_3_ (ppm)	Concentration of O_3_ by province, round the number to 4 places	Air Korea
CO (ppm)	Concentration of CO by province, round the number to 4 places	Air Korea
PM_10_ (μg/m^3^)	Concentration of PM_10_ by province	Air Korea

SO_2_, sulfur dioxide; NO_2_, nitrogen dioxide; O_3_, Ozone; CO, carbon monoxide; PM_10_, levels of particulate matter with aerodynamic diameter < 10 μm; KOSIS, Korean Statistical Information Service; KMA, Korea Meteorological Administration.

### Statical analysis and machine learning analysis

Many fields utilize machine learning^29^, and active research is underway in the health sector to utilize machine learning to analyze cancer survival^30^ and predict emergency room admission^31^. Furthermore, medical big data have been used to develop personalized medicine for dry eye disease^32^. In our study, conjunctivitis prevalence was set as a dependent variable, and meteorological, air quality, and demographic factors were independent variables. By analyzing prevalence patterns, influencing factors were identified and predictive modeling performed. In this process, exploratory data analysis (EDA) on each variable was conducted to examine each characteristic and identify its impact on prevalence. Finally, the relationship between prevalence and each variable was identified using traditional analysis methods, such as multiple linear regression analysis and machine learning analysis. Machine learning analyses included XGBoost, decision tree, and random forest methods. The total numbers of data sets for analysis were 1428. The machine learning analysis model was maintained at a 90% training set (number of set = 1288) and 10% test set (number of set = 140). The performance of each model was evaluated using RMSE values. The statistical analysis incorporated regression analysis to define correlation factors between independent variables. All statistical analyses were performed using R software (version 3.6.1). Statistical significance was defined as *P* < 0.05.

## Supplementary Information

Supplementary Information.

## Data Availability

The datasets generated during and/or analyzed during the current study are available from the crorresponding author upon reasonable request.
