# Supplementary material for: Correlation between air pollution and prevalence of conjunctivitis in South Korea using analysis of public big data
Source: Sci Rep. 2022 Jun 16;12:10091. doi: 10.1038/s41598-022-13344-5 (PMC9203752; doi:10.1038/s41598-022-13344-5)
Supplement: Supplementary file 1 — Supplementary Information. [file 41598_2022_13344_MOESM1_ESM.docx]

Supplementary Table 1. National Ambient Air Quality Standards of South Korea

| Air pollutant | National Air Quality Standards | |
| --- | --- | --- |
|  | Averaging time | Standard level |
| Sulfur dioxide (SO_2_) | 1-year | 0.02 ppm or under |
|  | 24-hours | 0.05 ppm or under |
|  | 1-hour | 0.15 ppm or under |
| Carbon monoxide (CO) | 8-hours | 9 ppm or under |
|  | 1-hour | 25 ppm or under |
| Nitrogen dioxide (NO_2_) | 1-year | 0.03 ppm or under |
|  | 24-hours | 0.06 ppm or under |
|  | 1-hour | 0.10 ppm or under |
| Particulate matter (PM_10_) | 1-year | 50 μg/m^3^ or under |
|  | 24-hours | 100 μg/m^3^ or under |
| Ozone (O_3_) | 8-hours | 0.06 ppm or under |
|  | 1-hour | 0.1 ppm or under |

PPM: parts per million

Monthly air quality standard for air environment standards is not established.
